# Meal replacement in dietary management of type-2 diabetes mellitus: a scoping review protocol

**DOI:** 10.1186/s13643-020-01517-0

**Published:** 2020-11-23

**Authors:** Lew Leong Chen, Arimi Fitri Mat Ludin, Suzana Shahar, Zahara Abdul Manaf, Noorlaili Mohd Tohit

**Affiliations:** 1 Biomedical Science Programme, Faculty of Health Sciences, Universiti Kebangsaan Malaysia, Jalan Raja Muda Abdul Aziz, 50300 Kuala Lumpur, Malaysia; 2 Center for Healthy Ageing and Wellness, Faculty of Health Sciences, Universiti Kebangsaan Malaysia, Jalan Raja Muda Abdul Aziz, 50300 Kuala Lumpur, Malaysia; 3 Dietetic Programme, Faculty of Health Sciences, Universiti Kebangsaan Malaysia, Jalan Raja Muda Abdul Aziz, 50300 Kuala Lumpur, Malaysia; 4 Department of Family Medicine, University Kebangsaan Malaysia Medical Centre (UKMMC), Cheras, 56000 Kuala Lumpur, Malaysia

**Keywords:** Scoping review, Meal replacement, Type-II diabetes mellitus, Glycemic control, Weight reduction

## Abstract

**Background:**

The prevalence of type-2 diabetes mellitus (T2DM) has been increasing globally. Without proper management, T2DM can develop into serious complications and even death. Diet modification is one of the most effective tools in managing T2DM at the early stage, but it requires knowledge and compliance from the patients. Thus, meal replacement (MR) has gained its popularity as a tool for diet modification to improve glycemic control and also reducing weight in T2DM patients. There are several existing meal replacement studies but not much is known on the general scope and effect of these existing MRs. Hence, this review is aimed to provide an overview of the existing evidences regarding the application of meal replacement on T2DM patients and identify the gaps or limitations in the studies.

**Methodology:**

The scoping review will be carried out in six stages: (1) identifying the research question, (2) identifying relevant studies through electronic databases (i.e., PubMed, Scopus, Cochrane Reviews, Google Scholar, EBSCOHOST, Science Direct) and also gray literature, and (3) selection of studies to be included based on inclusion criteria. Search and initial screening of studies to be included will be conducted by two independent reviewers. Discrepancies will then be solved through discussion with other reviewers; (4) charting and categorizing extracted data in a pretested data extraction form; (5) collating, summarizing, and reporting the results; and lastly, (6) conducting consultation with stakeholders and experts in diabetes.

**Discussion:**

This scoping review protocol is aimed to provide a framework enabling us to map and summarize the findings from existing studies involving meal replacement. It will help researchers to identify the research gap and provide recommendations for future meal replacement studies. The results from this scoping review will be useful to various stakeholders in healthcare. It is also part of a research project in which the information obtained will be utilized in a clinical trial of a developed meal replacement plan. Dissemination of knowledge will also be done through presentations at related scientific conferences.

**Supplementary Information:**

The online version contains supplementary material available at 10.1186/s13643-020-01517-0.

## Background

Diabetes mellitus is a global public health threat affecting people worldwide. The International Diabetes Federation (IDF) estimated that the global prevalence of diabetes mellitus is 451 million in 2017 and projected to increase to 693 million by 2045 [1]. Among them, over 90% of diabetes mellitus cases are type-2 diabetes mellitus (T2DM) [2]. Type-2 diabetes mellitus is a chronic metabolic disorder which is previously known as non-insulin-dependent diabetes mellitus. It is the most common form of diabetes mellitus characterized by hyperglycemia, insulin resistance, and relative insulin deficiency [3, 4]. Both genetic and lifestyle factors will lead to T2DM, with obesity, low physical activity level, poor dietary practices, smoking habits, and alcohol use being the primary risk factors for T2DM [5–7].

Without proper treatment or management, T2DM will develop into serious complications and lead to potentially fatal conditions. In the year 2012 alone, 1.5 million deaths worldwide were directly caused by diabetes [8]. There are also complications related to T2DM such as myocardial infarction, non-alcoholic fatty liver disease, and diabetic nephropathy [9, 10]. T2DM is widely regarded as an incurable but manageable condition through medications and lifestyle modifications. In the management of T2DM through pharmacological approach, medications such as insulin injection, biguanides, thiazolidinedione, sulfonylureas, meglitinides, and alpha-glucosidase inhibitors are used [11].

The first-line management for T2DM patients is lifestyle modification, specifically weight management for obese and overweight patients. The prevalence of obesity among patients with T2DM is high throughout the world. Over 30% of participants is reported with obesity in 38 of 44 studies pooled in a systematic review on observational studies for the prevalence of obesity in T2DM patients [12]. Excess weight in T2DM patients can lead to higher risks for additional complications such as cardiovascular diseases. On the contrary, weight loss has led to an improved 24 h plasma glucose profile, reduced HbA1c, and increased insulin sensitivity [13]. The American Diabetes Association also recommended maintaining > 5% weight loss for patients with T2DM who are overweight or obese through diet, exercise, and behavioral therapy [14]. This is supported by the outcome in the DiRECT study has shown a clinically significant 46% remission to non-diabetic state among 306 participants who lost up to 15 kg of weight [15].

Dietary intervention is effective to achieve targeted weight and glycemic control in T2DM patients [16]. However, this approach can be challenging for patients and also healthcare providers as it requires compliance from the patients to follow the guidance provided to them. It might also be an effortful task for the patients as it requires a certain level of self-care knowledge and skills to prepare their own meals. The diabetes education given to the patients might be insufficient to help them develop self-efficacy skills for managing their diet [17]. Time limitation, family responsibilities, and busy schedules may also hinder patients from preparing their own meals based on suggestions provided by dietitians [17–19].

Meal replacements (MR) are prepackaged food products or drinks that are designed to replace one or more meals and provide a defined amount of energy [20]. It is found out that portion-controlled MR is able to produce 31.5% more clinically significant weight loss (> 5% weight loss 1 year) and significant BMI reduction in obese subjects as compared to standard food-based plans [21]. In the Look AHEAD study involving 5145 overweight or obese participants with T2DM, meal replacement as a part of intensive lifestyle intervention has reduced HbA1c significantly after 1 year intervention period [22, 23]. Diet planning through meal replacement products is a useful approach for T2DM patients as they can be delivered easily to the community without much help from healthcare professionals. It has also proven to be a safe and effective method for increasing dietary compliance [24]. There are various MR trials and researches being carried out to date, but not much is known on the thorough review of type, composition, dosage, and delivery. There is also a lack of review on risks or side effects of MR on T2DM patients. Although MR has shown to be an effective intervention in managing T2DM patients, there are studies that have shown that MR comes with side effects and risks. A cohort study by Johansson on 8361 participants shown that MR use has increased risks for symptomatic gallstones that require cholecystectomy [25]. Certain meal replacement liquid does not contain enough fiber which may lead to undesired effects in the bowel system if consumed daily. Hence, this review is being carried out to map out this information as they are important in continuous quality improvement of MR and potentially leading to better innovation.

With this scoping review, we aim to collate literature on meal replacement for the dietary management of T2DM patients. We will examine meal replacements available for type-2 diabetes mellitus patients and their effects, respectively, on glucose control/HbA1C, weight reduction, and other health status of the patients. The risks or side effects from the meal replacements will also be identified. The findings will be mapped by categorizing the papers and summarizing them. Specifically, the purposes of conducting this scoping review are as follows:

i) To provide an overview of existing studies regarding the usage of a self-administered meal replacement on adult type-2 diabetic patients.

ii) To map out the outcomes of a self-administered meal replacement on specific parameters (Hba1c, glucose, and weight reduction) of the participants.

iii) To map out the side effects of self-administered meal replacement among adult type-2 diabetic patients.

## Aim

The aim of this manuscript is to present a protocol for a scoping review of self-administered meal replacements in managing adult type-2 diabetes mellitus patients.

## Methodology

The purpose of this scoping review is to gain an overview of meal replacements and its outcome in managing patients with T2DM. Scoping review is identical to systematic review but differs in purpose as scoping review aims to comprehensively map the evidences of a topic while systematic review seeks to summarize and analyze the most ideal topic on a specific question [19].

The methods of this scoping review will be based on Arksey and O’Malley’s seminal framework for scoping reviews [19]. The approach to searching, screening, and reporting of scoping review as suggested by Levac, Colqohoun, and O’ Brien will be modified and utilized [26]. We will also use PRISMA-P as a checklist for aspects applicable to the preparation of a scoping review protocol [27]. A completed PRISMA-P checklist is attached to this protocol (Additional file 1). The guidelines recommended in PRISMA Extension for Scoping Reviews (PRISMA-ScR) checklist will be followed when reporting the scoping review [28].

There are six stages involved in the scoping review framework which included (1) identifying the research question; (2) identifying relevant studies; (3) selection of studies to be included; (4) charting of information and data from the studies; (5) collating, summarizing, and reporting the results; and (6) conducting consultation with stakeholders and experts in diabetes.

### Stage 1: Identifying research questions

1. What are the types, composition, dosage, delivery, and duration of meal replacement plans available for managing T2DM patients?
2. What are the outcomes of a meal replacement on glycemic control/HbA1C, weight reduction, and other health status of T2DM patients?
3. What are the side effects experienced by T2DM patients taking meal replacements?

### Stage 2: Identifying relevant studies

Identification of studies relevant to this review will be achieved by searching for studies published between January 2000 and August 2020. The following PICO (population, intervention, , and outcomes) as shown in Table 1 below is used to help us identify relevant evidence to be included in the study.

**Table 1:** Table of PICO

**Population**	This review will focus on adults with type-2 diabetes mellitus. Studies reporting on adult T2DM patients aged more than 18 years old will be included. Intervention including mixed population age will be included only if the data can be separated. There will be no restriction on the severity of T2DM of subjects in the study.
**Intervention**	Any intervention or program utilizing self-administered meal replacements will be considered. A meal replacement is defined as prepackaged food products, drinks, or meals that are designed to replace one or more meals and provide a defined amount of energy [20].
**Comparator**	No comparator is required for this review.
**Outcome**	Any studies that report the outcome related to the health status of the T2DM subject, including glycemic control, weight reduction, or side effects.

The search will be conducted through several electronic databases (i.e., PubMed, Scopus, Cochrane Reviews, Google Scholar, Ebscohost, Science Direct, Web of Science). The search for online databases will be filtered for humans’ and adults’ results. The results will be downloaded into EndNote and duplicate results will be removed [29]. All reference lists of included studies and other reviews that we came across during database searching will be screened through to identify any additional eligible relevant studies to be included in our review.

We will also search for gray literature (local non-indexed journals, websites, and theses) regarding meal replacement in managing patients with type-2 diabetes mellitus published within the same period. We will conduct a search through the first 20 pages of Google Search Engine to search for any related websites, non-indexed journals, research reports, or articles. Searches will also be done on gray literature databases (Open Grey, Grey Literature Report, EMBASE Conference Abstracts, and Web of Science Conference Proceedings Citation Index) to identify relevant reports or conference proceedings. Relevant health and diabetes organization websites including the World Health Organization, American Diabetes Association, International Diabetes Federation, Diabetes UK, Joslin Diabetes Center, Ministry of Health Malaysia Virtual Library, and Malaysia National Diabetes Institute will also be searched for resources related to the review. Apart from manual searching in the thesis room of the National University of Malaysia, library database and online local thesis database will also be searched to identify dissertations/theses that are applicable to this review.

Two independent reviewers (LC and AF) will be performing the search in parallel using a comprehensive search strategy. Search terms from PICO, key words, subject headings, and synonyms such as meal replacement, type-II diabetes mellitus, glycemic control, weight reduction, and risks will be generated by the research team members in order to capture any potential resources from the databases. Table 2 outlines the initial keywords and search terms generated. Boolean operators (AND, OR, NOT) will be used to combine search terms within related keywords and is adapted to the syntax used by each database. If there are any search terms missing from the initial search terms, an additional search will be carried out using the updated search terms. Table 3 shows the search strings generated.

**Table 2:** List of keywords and synonyms generated as search terms

Meal replacement	Type-2 diabetes mellitus	Glycemic control	Weight reduction	Risks
Food substitute	Diabetic	HbA1c	Weight loss	Side effect
Alternative serving	Diabetes	Random blood sugar	Weight reduction	Danger
Diet alternative	Non-insulin-dependent diabetes mellitus	Glucose	Decrease in body weight	Hazardous
Alternative nutrition	Diabetes mellitus-onset	Fasting blood sugar	Body weight changes	Adverse effect
Diet replacement	Type-II diabetes mellitus	2-h post-prandial blood glucose	BMI reduction	Bad
Diet plan exchange		Glucose tolerance	Fatness	Negative effect
Replacement drink			Adiposity	Harmful
Alternate nutrition				Detrimental
Oral nourishing supplement				Antagonistic
Medicinal food				Unsafe

**Table 3:** List of search strings

Search string 1	“Meal Replacement*” OR “Food Substitute*” OR “Alternative Serving*” OR “Diet Alternative*” OR “Alternative Nutrition” OR “Diet Replacement*” OR “Diet plan Exchange” OR “Replacement Drink*” OR “Alternate nutrition” OR “Oral nourishing supplement*” OR “Medicinal food” AND “Type-2 Diabetes Mellitus” OR Diabetic OR Diabetes OR “Non-insulin dependent Diabetes Mellitus” OR “Diabetes Mellitus-onset” OR “Type-II Diabetes Mellitus”
Search string 2	“Meal Replacement*” OR “Food Substitute*” OR “Alternative Serving*” OR “Diet Alternative” OR “Alternative Nutrition” OR “Diet Replacement*” OR “Diet plan Exchange” OR “Replacement Drink*” OR “Alternate nutrition” OR “Oral nourishing supplement*” OR “Medicinal food” AND “Type-2 Diabetes Mellitus” OR Diabetic OR Diabetes OR “Non-insulin dependent Diabetes Mellitus” OR “Diabetes Mellitus-onset” OR “Type-II Diabetes Mellitus” AND “Glycaemic control” OR HbA1c OR “Random Blood Sugar” OR Glucose OR “Fasting blood sugar” OR “2-Hour Post-Prandial Blood Glucose” OR “Glucose tolerance”
Search string 3	“Meal Replacement*” OR “Food Substitute*” OR “Alternative Serving*” OR “Diet Alternative” OR “Alternative Nutrition” OR “Diet Replacement*” OR “Diet plan Exchange” OR “Replacement Drink*” OR “Alternate nutrition” OR “Oral nourishing supplement*” OR “Medicinal food” AND “Type-2 Diabetes Mellitus” OR Diabetic OR Diabetes OR “Non-insulin dependent Diabetes Mellitus” OR “Diabetes Mellitus-onset” OR “Type-II Diabetes Mellitus” AND “Weight reduction” OR “Weight Loss” OR “Weight reduction” OR “Decrease in body weight” OR “Body Weight Change*”
Search string 4	“Meal Replacement*” OR “Food Substitute*” OR “Alternative Serving*” OR “Diet Alternative” OR “Alternative Nutrition” OR “Diet Replacement*” OR “Diet plan Exchange” OR “Replacement Drink*” OR “Alternate nutrition” OR “Oral nourishing supplement*” OR “Medicinal food” AND “Type-2 Diabetes Mellitus” OR Diabetic OR Diabetes OR “Non-insulin dependent Diabetes Mellitus” OR “Diabetes Mellitus-onset” OR “Type-II Diabetes Mellitus” AND “Risk*” OR “Side effect” OR “Dangerous” OR “Hazardous” OR “Adverse effect*” OR “Bad” OR “Negative effect*” OR “Harmful” OR “Detrimental” OR “Antagonistic” OR “Unsafe”

### Stage 3: Selection of studies to be included

The inclusion criteria for the search will be studies ranging from January 2000 to August 2020 related to meal replacement with T2DM affected patients aged 18 and above (adult and elderly). These will include articles from primary studies, technical reports, and review articles. Gray literatures (i.e., Websites, local non-indexed journals, and theses) will also be included. Studies from all countries will be considered, but studies that are not available in English and Malay will be excluded. Studies will also be excluded if they include (1) type-1 diabetes mellitus patients, (2) pregnant women, (3) pediatric populations, (4) are not human studies, and (5) meal replacements that are not self-administered. Complete inclusion and exclusion criteria are as described in Table 4.

**Table 4:** Inclusion/exclusion criteria for study selection

Inclusion criteria	Exclusion criteria
Studies ranging from January 2000 to Aug 2020 related to meal replacement with T2DM-affected patients aged 18 and above (adult and elderly).	Studies that are not available in English and Malay language
	Studies with type-1 diabetes mellitus patients
	Studies with pregnant women
	Studies which are not human studies
	Studies which meal replacements that are not self-administered

Screening and selection of articles will be carried out in a three-step process. In the first step, two researchers (LC and AF) will screen through all titles from the search results in databases and gray literatures using the search term generated from stage two previously. All articles involving meal replacements and T2DM patients will be considered.

In the second step, all researchers (LC, AF, SS, ZAM, and NMT) will be working independently to screen through the selected titles and abstract obtained from step 1 for articles potentially related to the objectives. Researchers will meet up to compare the results and to resolve discrepancies. A diabetes professional will be consulted if the discrepancies cannot be resolved.

In the third step, similarly, all researchers (LC, AF, SS, ZAM, and NMT) will be working independently to screen through the full article obtained from the second step. Efforts will be made to obtain the full papers through the university library if full papers are not available online. The full articles will be studied through to ensure that they meet the objectives. Researchers will meet upon completion to compare results and resolve any discrepancies.

Articles which are irrelevant to the objectives will be excluded, and the results of relevant articles from the search will be managed by Endnote X7 program and its extracted data will be recorded in Microsoft Excel. The entire process of reviewing will be guided by using the PRISMA-ScR (PRISMA Extension for Scoping Reviews) checklist.

### Stage 4: Charting of information and data from the studies

A data charting table (Additional file 2) is developed by the research team members to confirm the relevance of studies screened and to extract findings from the studies. Findings will be extracted and categorized into a table of evidences as below:

A. Author(s)

B. Publication year

C. Objectives or purposes of the study

D. Participants’ characteristics – age, BMI

E. Study design

F. Settings

G. Meal replacement type

H. Dosage and calorie

I. Control groups (if any)

J. Duration

K. Effect on HbA1c and glucose

L. Effect on weight reduction

M. Effect on other health status

N. Side effects

Other additional findings which are not included in the category of charting table are listed under an extra column labeled as “notable findings.”

The main outcome of the data will be the type and dosage of meal replacement and its effect on HbA1c, glucose, and weight reduction. An additional outcome will be the side effects of the meal replacement.

The charting table will be discussed by all researchers in a meeting and pretested before the implementation of 10 articles to ensure the table captures significant information correctly. Two researchers (LC and AF) will then work independently to extract and chart the data from articles that are screened previously. The other researchers (SS, ZAM, NMT) will check the extracted data for accuracy. Any disagreements will be discussed during group meetings.

### Stage 5: Collating, summarizing, and reporting of results

The literature will be mapped and arranged with the concepts mentioned previously. The results of the data extracted will be summarized and analyzed. The general characteristics of the included studies will be summarized. Descriptive statistics such as percentages or frequencies will be used to provide summary characteristics of the studies based on the type of meal replacement plan being used in the studies. The studies will be categorized based on their study design. Data will be presented using a table of findings based on the effects of the meal replacement on glycemic control parameters and weight reduction. Risks and side effects of meal replacement products on T2DM patients will also be discussed. Limitations and research gaps for the studies will also be discussed to provide better recommendations for future meal replacement studies.

### Stage 6: Consultation with stakeholders

Consultation with stakeholders and experts are optional but recommended in a scoping review. The expert will be able to provide assistance on finding relevant articles and also give extra feedback on the results and data analyzed. We will present our findings at local-related conferences. Feedback from the audiences will be recorded and used to supplement our findings.

### Patient and public involvement

As this scoping review only involves literature-based studies, there is no patient or public involvement.

## Discussion

The aim of this scoping review protocol is to provide a framework enabling us to review the research foci on meal replacement for the past 20 years. It will help us to map and summarize the findings from existing researches to identify limitations and provide recommendations for future research in nutrition intervention for diabetes. Although there have been several numbers of meal replacement studies carried out, there are no comprehensive evidences from these existing studies to provide us an overview on the current meal replacement scopes. It is anticipated that the result will be useful to a variety of stakeholders. The target audiences for the result will be those who are involved in diabetes and obesity healthcare sectors, including pharmaceutical industries, institutional decision makers, and researchers. No ethics approval is required as this study only involves reviewing and extracting data from readily available publications and materials.

This review will be part of a research project in which the information obtained will be utilized in a clinical trial of a developed meal replacement plan. The findings of this review will be summarized and written as an article for peer-reviewed publication. Our approaches to knowledge dissemination will also be done through presentations at T2DM and obesity-related conferences such as the Diabetes Asia Conference.

## Supplementary Information

**Additional file 1.** PRISMA-P 2015 Checklist

**Additional file 2.** Data extraction form

## Data Availability

The datasets used and/or analyzed during the current study are available from the corresponding author on reasonable request.

## Abbreviations

T2DM: Type-2 diabetes mellitus
MR: Meal replacement
PRISMA-P: Preferred reporting items for systematic reviews and meta-analyses - protocol
PRISMA-ScR: Preferred reporting items for systematic reviews and meta-analyses - scoping reviews
