# Supplementary material for: Meal replacement in dietary management of type-2 diabetes mellitus: a scoping review protocol
Source: Syst Rev. 2020 Nov 23;9:265. doi: 10.1186/s13643-020-01517-0 (PMC7686729; doi:10.1186/s13643-020-01517-0)
Supplement: Supplementary file 2 — Additional file 2. Data extraction form [file 13643_2020_1517_MOESM2_ESM.docx]

| **Study** | **Author** | **Year** | **Purpose** | **Participants Characteristics (Diagnosis and Age)(Sample Size)** | **Study design** | **Settings** | **Meal Replacement type** | **Dosage and calorie** | **Control Groups (if any)** | **Duration** | **Effect on HbA1c and glucose** | **Effect on weight reduction** | **Effect on other health status** | **Side effects** | **Notable Findings** |
| --- | --- | --- | --- | --- | --- | --- | --- | --- | --- | --- | --- | --- | --- | --- | --- |
|  |  |  |  |  |  |  |  |  |  |  |  |  |  |  |  |

**Data Extraction Form**
